# Contact Unmodified Antisense DNA Biotechnology (CUADb)-Based Oligonucleotide Insecticides and RNA Biocontrols: Molecular Bases and Potential in Plant Protection

**DOI:** 10.3390/cimb48020235

**Published:** 2026-02-23

**Authors:** Vol Oberemok, Kate Laikova, Jamin Ali, Ilyas Chachoua, Nikita Gal’chinsky

**Affiliations:** 1 Department of General Biology and Genetics, Institute of Biochemical Technologies, Ecology and Pharmacy, V.I. Vernadsky Crimean Federal University, Simferopol 295007, Russia; botan_icus@mail.ru (K.L.); pcr.product@gmail.com (N.G.); 2 College of Plant Protection, Jilin Agricultural University, Changchun 130118, China; j.alirana@yahoo.com; 3 Department of Molecular Biology and Genetics, Bilkent University, Ankara 06800, Turkey; ilyas.chachoua@bilkent.edu.tr

**Keywords:** oligonucleotide insecticides, CUAD biotechnology, ‘genetic zipper’ technology, DNA containment, RNA biocontrols, double-stranded RNA technology, RNA interference, next-generation insecticides, plant protection

## Abstract

Recent advances in molecular genetics, nucleic acid synthesis, and bioinformatics have provided novel opportunities for plants’ protection against insect pests. Currently, both DNA and RNA serve as active insecticidal ingredients, transcending their traditional role as carriers of genetic information. This novel activity is achieved through two fundamentally distinct mechanisms. The first one is DNA containment (DNAc), employing oligonucleotide insecticides based on contact unmodified antisense DNA biotechnology (CUADb), also known as ’genetic zipper’ technology. The second one is RNA interference (RNAi), employing RNA biocontrols based on double-stranded RNA (dsRNA) technology. The investigation of the molecular mechanism underlying the antisense activity of nucleic acids emerged in the early 1960s. While the antisense effects of RNA in gene silencing through interference (RNAi) was documented in the late 1990s as antiviral immune responses in nematodes, the CUADb antisense approach initially emerged as a powerful strategy for pest control against lepidopterans in 2008. The CUADb approach relies on disrupting rRNA biogenesis and ribosome production, while RNAi shows the best results in mRNA degradation and no efficient result is known for rRNA. The efficacy of these approaches appears to be species dependent. For example, CUADb demonstrates optimal activity against Sternorrhyncha (e.g., aphids, mealybugs, psyllids, and scale insects), thrips, and mites. In turn, the RNAi strategy shows a strong insecticidal potential against beetles from the Tenebrionidae and Chrysomelidae families. Here, we will review the differences between the two technologies, their mechanisms of action and the current challenges facing their adoption.

## 1. Introduction

The relentless innovation in insect pest control drives the continuous replacement of older chemistries (carbamates and organophosphates) by contemporary agents (neonicotinoids and diamides) [1,2,3]. Yet the intractable challenge of genetic resistance ensures this cycle persists [3,4], demanding insecticides with extended utility, heightened selectivity, enhanced biodegradability, and a reduced carbon footprint. Biomolecules have recently emerged as promising tools that demonstrate high potential in overcoming these challenges [5,6,7,8]. Both DNA and RNA have shown remarkable insecticidal effects, enabled by advances in molecular genetics, synthesis, and bioinformatics [9,10,11,12]. This has led to the development of two distinct technologies: 1. oligonucleotide insecticides based on contact unmodified antisense DNA biotechnology (CUADb), also known as ‘genetic zipper’ technology (a DNA–RNA duplex formed by 11-mer antisense oligonucleotide with target sequence of the pre-rRNA/rRNA of an insect pest followed by the subsequent degradation of target rRNA by RNase H) [13], and 2. RNA biocontrols utilizing double-stranded RNA (dsRNA) technology [8,14]. Along with CRISPR/Cas technology, which is the main gene editing tool used to control insect pests [15], these technologies together represent the three main antisense ap-proaches to insect pest control, each using unique molecular mechanisms. RNAi functions through guide RNA-mRNA duplexes cleaved by Argonaute nuclease, while CUADb operates via guide DNA-rRNA duplexes processed by DNA(-RNA hybrid)-guided RNase, such as RNase H1 [15]. Antisense technologies provide taxon-specific efficacy enabling combinatorial strategies; their divergent molecular targets preclude universal application, increasing the chance of efficiently targeting different organisms.

After two decades of development, RNAi still lacks a standardized design algorithm for reliable insecticide development. Conversely, CUADb possesses a validated design framework [16] with potential efficiency against no less than 10–15% of all insect pests, demonstrating particular efficacy against Sternorrhyncha (e.g., aphids and mealybugs) and superorder Paraneoptera in general [17], whereas RNA biocontrols show the strongest activity mainly against Coleoptera. These independent ‘fraternal twins’, dsRNA technology and ‘genetic zipper’ technology (CUADb), hold synergistic potential for developing highly selective, next-generation pest control agents. This review addresses the paradigm shift toward nucleic acid-based solutions, with dedicated focus on CUADb as an innovative technology characterized by its novel DNAc mechanism and advanced insecticidal profile.

## 2. CUADb-Based Oligonucleotide Insecticides

In 2008, our research group demonstrated the use of short unmodified antisense DNA oligonucleotides as contact insecticides [18]. Initial work on the spongy moth (*Lymantria dispar*) demonstrated that effective gene silencing using oligonucleotide insecticides (oligoRING and oligoRIBO-11) depended critically on the target gene and its expression levels [19,20]. Subsequent research identified rRNA as a prime target, leading to the development of oligonucleotide insecticides based on 11-mer DNA sequences complementary to pest rRNA in 2019 [15]. rRNA is an optimal target due to its abundance (constituting ~80–85% of cellular RNA) and the substantial energy investment (>60% of cellular energy) required for ribosome production and maintenance [21,22]. Insect rRNA comprises 28S rRNA (~3900 nt), 18S rRNA (~1920 nt), 5.8S rRNA (~160 nt), 5S rRNA (~120 nt), and mitochondrial rRNAs including 16S rRNA (~1140 nt) and 12S rRNA (~600 nt) [21]. Ribosomal RNA (rRNA) serves as the foundational framework of the ribosome, a vital organelle orchestrating protein synthesis. Unlike messenger RNA (mRNA), which carries genetic instructions for protein assembly, rRNA is a non-coding RNA that catalyzes the actual process of protein synthesis within the ribosome; this involves pairing with mRNA and tRNA, maintaining the structure of ribosome, and the formation of peptide between amino acids [23,24]. The first successful rRNA targeting with unmodified antisense DNA oligonucleotides was achieved against 5.8S rRNA of *L. dispar* [19]. CUADb-based oligonucleotide insecticides are designed using the DNAInsector program (dnainsector.com) or manually via GenBank sequences of pest pre-rRNA and mature rRNA. Synthesis employs the phosphoramidite method through liquid-phase or solid-phase synthesis using instruments such as the ASM-800 (BIOSSET, Russia), OligoPilot™ (Cytiva, Sweden), PolyGen 10-Column DNA Synthesizers, and others [25]. One of market leaders in liquid phase synthesis of DNA, Sumitomo Chemical Co., Ltd. (Tokyo, Japan), offers the synthesis of 1 kg of unmodified oligonucleotides 11 nt long for 25,000 USD [26].

Oligonucleotide insecticides demonstrate high efficacy against sternorrhynchans (e.g., aphids, mealybugs, psyllids, and scale insects), thrips, and mites, with successful targeting documented across multiple species: 28S rRNA in *Unaspis euonymi*, *Dynaspidiotus britannicus*, *Icerya purchasi*, *Ceroplastes japonicus*, *Aonidia lauri*, and *Coccus hesperidum* [6,8,17,25,27,28]; 18S rRNA in *Pseudococcus viburni* [29]; mitochondrial 16S rRNA [30]; ITS2 regions in *Macrosiphoniella sanborni*, *Schizolachnus pineti* [26,31], and *Trioza alacris* [13]; and also *Tetranychus urticae* [32], demonstrating high insecticidal potential for oligonucleotide acaricides [15]. A single contact treatment at 100 ng/μL (1 mg of DNA per m^2^ of foliage in 10 mL of the solution or 10 g/ha in 100 L) typically achieves approximately 80% mortality in pests within 3–14 days [11,33]. Detailed recommendations for the use of oligonucleotide pesticides are presented in the article by Oberemok et al. [34], as well as in other research articles on this research topics [8,13,17,32]. While highly effective against hemipterans and moderately effective against lepidopterans including *L. dispar*, their efficacy is notably lower against coleopterans such as *Leptinotarsa decemlineata* and requires further investigation of their resistance to this approach [35]. The 11-mer length provides species specificity with a uniqueness frequency exceeding 1/4.19 × 10^6^, covering most agricultural applications [6]. Moreover, previous studies of the effect of oligonucleotide insecticides on the biochemical parameters of the plants *Quercus robur* L., *Malus domestica* Bokh [36], and *Triticum aestivum* L. [37,38], and on the viability of the insects *Manduca sexta* L., *Agrotis ipsilon* Hufnagel [39], and *Galleria mellonella* L. [20], showed their safety for non-target organisms. However, it was assumed that non-canonical base pairing, such as A:C (C:A) and G:U (T:G) [8,31,40,41], may occur between oligonucleotide insecticides and the imperfect sites of rRNAs and lead to non-specific effects. Definitely, non-canonical base pairing should be taken into consideration during the design of oligonucleotide insecticides so as not to harm non-target organisms [8]. rRNA’s dual advantages of abundance and inter-species variability make it superior to less concentrated cellular mRNAs as a target. Contact delivery (CUADs) outperforms oral delivery (ODUADs) because hemipterans possess digestive DNases and extra-oral salivary barriers that interfere with oral delivery [42].

**Figure 1:**
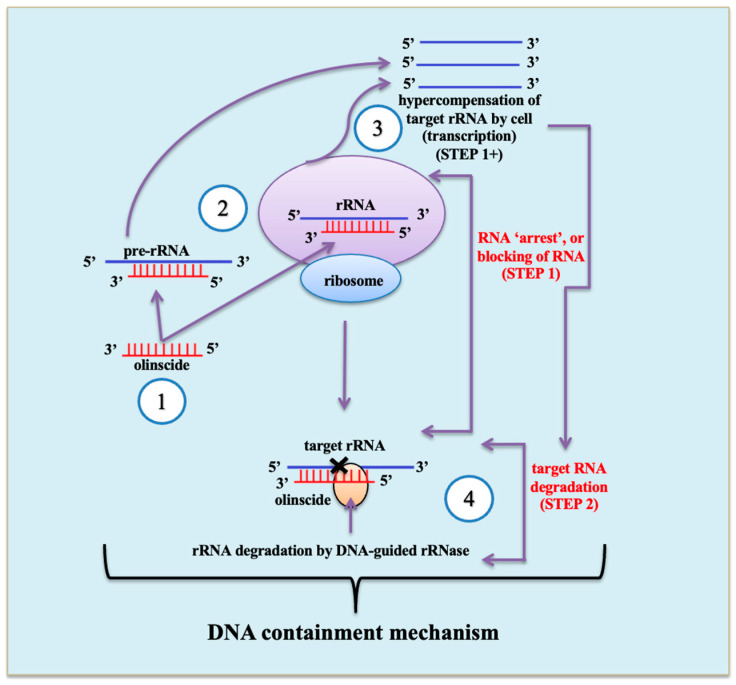
DNA containment mechanism (DNAc) for insect pest control. (1) Antisense DNA oligonucleotides complementary to pest mature rRNA or pre-rRNA are applied as insecticides. (2) Duplex formation occurs between the oligonucleotide and target rRNA/pre-rRNA. (3) In the first step of DNAc, antisense DNA ‘arrests’ rRNA or pre-rRNA, leading to hypercompensation and containment of ribosome biogenesis (‘arrested ribosomes’). (4) In the second step, DNA(-RNA hybrid)-guided RNase cleaves the target rRNA/pre-rRNA, reducing its concentration.

The 2008 discovery of contact oligonucleotide insecticides was unexpected, contradicting established views that unmodified oligodeoxyribonucleotides are rapidly degraded by DNases [43] and that rRNA resists antisense DNA-mediated degradation [44,45]. Previous research has focused exclusively on gene downregulation, overlooking insecticidal potential [18,43]. Oligonucleotide insecticides refuted these assumptions through a two-step DNA containment (DNAc or ‘genetic zipper’) mechanism (Figure 1; left part): initial-target rRNA ‘arrest’ halts ribosome function and triggers rDNA transcription hypercompensation, followed by degradation of the target rRNA by DNA(-RNA hybrid)-guided RNases, such as RNase H1 [8,15]. This ‘genetic zipper’ mechanism [13] induces metabolic shifts toward lipid-based energy synthesis, enhancing the biogenesis of ribosomes and ATP production in mitochondria. Ultimately, widespread kinase downregulation—including mTOR, which regulates ribosome biogenesis through mTORC1—causes ‘kinase disaster’ due to ATP insufficiency, while significant RNase H1 upregulation occurs throughout DNAc [17]. Of note, such patterns of gene expression in insect cells have not been observed before for any of exogenous triggers causing different nucleolar stress responses known from scientific literature.

In 2011, three years after this discovery, Wang et al. [46] adapted the contact concept for dsRNA insecticides. Crucially, DNAc downregulates key RNAi enzymes (DICER1, Argonaute 2, and DROSHA), underscoring its mechanistic distinction from RNAi [17]. Thus, ssDNA in CUADb and dsRNA in RNAi represent fundamentally distinct antisense technologies and suggest antagonism between the two mechanisms, though with possible synergistic potential in pest management [15] (Table 1).

A working hypothesis proposes that DNA and RNA viruses (using their complementary genome sequences) may exploit the rRNA gene expression hypercompensation observed during the initial step of the DNA containment mechanism to increase the cellular ribosome numbers essential for viral replication [17]. Bioinformatics studies indicate that the viral hijacking of host genes is common [51]. Given rRNA’s dominance (~80% of cellular RNA) and the significant energy investment (>60%) in ribosome production [21,22], DNA viruses likely co-opted rRNA-like sequences during co-evolution [52] but their function was unknown, and based on obtained experimental data we suppose involvement of these viral rRNA-like sequences in triggering of rRNA synthesis (ON/OFF principle explained in [17]). Also, as a hypothesis that requires further investigation, it is likely that cells can produce their own antisense DNA (in a manner similar to Okazaki fragments, recruiting DNA-dependent DNA polymerases and RNA primers to initiate synthesis [53]) for regulation of rRNA synthesis and gene expression in general. Of note, for RNA viruses DNAc mechanism may also play an important role. Bioinformatically we found rRNA-like sequences near sequences of RNA-dependent RNA polymerases in genomes of some RNA viruses (for example, long fragment of human 28S rRNA was found by us in Longquan *Niviventer fulvescens* orthohantavirus genome (GenBank: PP211360.1) and in many other viruses). In this case complementary rRNA-viral RNA interactions may also boost production of host rRNA required for viral replication. Proximity of rRNA-like sequences to RNA-dependent RNA polymerase (crucial for virus replication) gene in virus genomes suggests parallels with compact layout of genetic systems in genomes like CRISPR/Cas system in genomes of bacteria [15].

From a practical point of view, targeting conserved regions of insect pest rRNA genes for oligonucleotide insecticide design delays target sites’ resistance development, as mutations occur less frequently in these areas [20,54]. When resistance develops, new effective CUADb insecticides could be designed by shifting the target site adjacent to the resistant region within the rRNA genes [8]. For example, many articles indicate that the most resistant pests are mites [55,56,57] and hemipterans [58,59,60,61]. Therefore, given that oligonucleotide pesticides have demonstrated their effectiveness against these pests [8,17,30,32], it was assumed that this technology would be effective against approximately 30% of the most resistant arthropods [62], showing substantial activity against Sternorrhyncha and spider mites [15,33]. As an additional advantage, it is also possible to create a cocktail of ssDNA fragments targeting multiple sites of rRNAs, thereby making it extremely hard for pests to develop a resistance to such ‘oligo cocktails’, as the probability of simultaneous mutations in the target sites is extremely low.

After their action, oligonucleotide insecticides undergo rapid and clean biodegradation in the environment (soils, water, and plants) via abiotic factors (temperature, pH, salinity, and UV radiation) and biotic factors (microbes and extracellular enzymes) [16,63]. Deoxyribonucleases in tissue homogenates of *Lymantria dispar*, *Leptinotarsa decemlineata*, *Icerya purchasi*, *Dynaspidiotus britannicus*, and *Aonidia lauri* and their host plants (*Quercus pubescens*, *Solanum tuberosum*, *Pittosporum tobira*, and *Laurus nobilis*) degrade oligonucleotide insecticides within 24 h at 27 °C [8,20,25,35], while *Macrosiphoniella sanborni* nucleases achieve degradation within 1 h [30], and modern DNA synthesis based on phosphoramidite chemistry minimizes greenhouse gas emissions (nitrogen oxide, methane, and carbon dioxide) compared to solid-phase methods [25], addressing climate concerns.

In conclusion, overcoming widespread pesticide resistance requires the development of novel and highly specific pesticide classes to reduce environmental impact. Oligonucleotide insecticides represent a remarkable advancement, using unmodified antisense oligonucleotides. Mirroring the trajectory of therapeutic oligonucleotides—which overcame early challenges through sustained research and investment [64]—CUADb-based pesticides offer selective action, rapid biodegradation, and increasingly cost-effective production, presenting a highly promising approach [65]. Based on our estimations, the ‘genetic zipper’ technology (CUADb) can potentially control no less than 15% of all insect pests using a simple and flexible algorithm [13]. Oligonucleotide insecticides can be designed using DNAInsector program (dnainsector.com, accessed on 10 February 2026) or manually using sequences of pest pre-rRNA and rRNA found in the GenBank database (https://www.ncbi.nlm.nih.gov/genbank/, accessed on 10 February 2026). It is now feasible for an individual to manually construct any oligonucleotide insecticide matching the pre-rRNA or rRNA sequences of sternorrhynchans, with a strong probability of its high efficacy. Notably, if insecticide resistance occurs, different strategies can be applied. Generally, new oligonucleotide insecticides can be created, displacing the target site to the left or to the right from the oligonucleotide insecticide resistance site of the rRNA or pre-rRNA [8]. Formulation additives (spreaders, adhesives, penetrators, and UV protectants) may enhance efficacy for other insect orders, pending environmental safety assessments. Also, optimized oligonucleotide design algorithms and multi-target site combinations can improve the performance of CUADb-based pest control agents. Future research will undoubtedly uncover deeper mechanistic details of DNAc and its potential. CUADb paves the way for sustainable, xenobiotic-free agriculture, positioning oligonucleotide pesticides as prospective cornerstone agents in pest control [13]. After almost two decades of research and substantial investments, ‘genetic zipper’ technology is now a fascinating and rapidly developing approach. Notably, CUADb based on antisense oligonucleotides has gained popularity and distribution for crop protection. For example, there have been developments using a similar protection approach based on modified antisense oligonucleotides, called the 2′-deoxy-2′-fluoro-D-arabinonucleic acid (FANA) approach [66,67], and the phosphorothioate modification of antisense oligonucleotides [68]. It is not unrealistic to believe that ASO-based pesticides may become a reality sooner rather than later, advancing more quickly by leapfrogging past existing technical solutions and leading to first commercially available pesticides based on antisense 11-mer oligonucleotides in the very near future [64].

Another nucleic acid-based technology for plant protection is based on dsRNA, with its own key peculiarities (Table 1), advantages and challenges. The application of double-stranded RNA (dsRNA) for the sequence-specific degradation of targeted mRNA via RNAi is emerging as an important tool for the development of novel RNA-based sustainable insect management strategies and will be discussed below.

## 3. RNA Biocontrols

**Figure 2:**
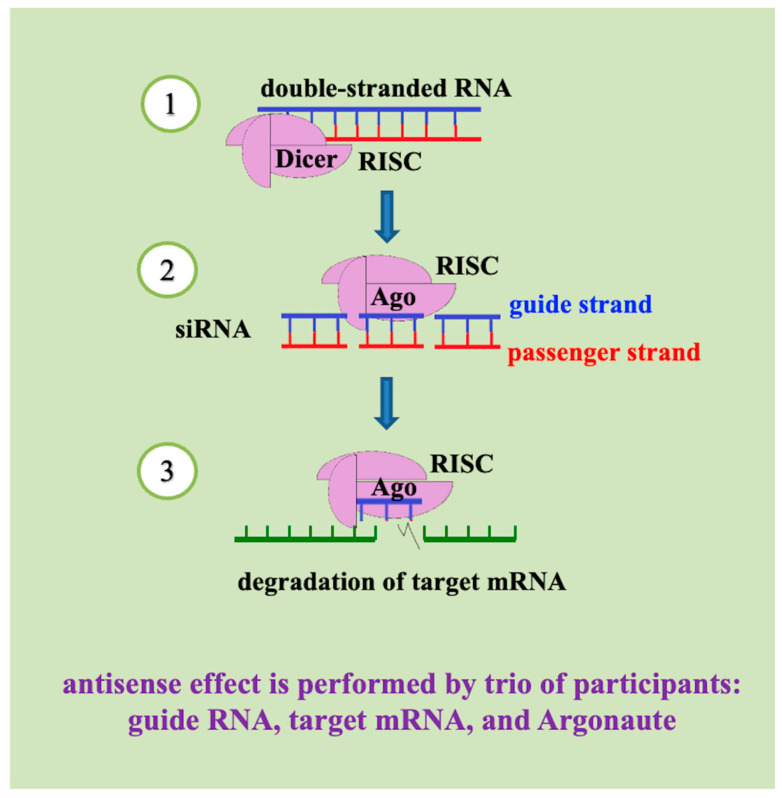
Main pathway of RNA interference (RNAi) utilized for the development of RNA-based biocontrols. (1) Double-stranded RNA (dsRNA) is processed by Dicer into siRNAs, which are incorporated into the RNA-induced silencing complex (RISC). (2) The guide strand directs Argonaute (Ago) to complementary target mRNA, (3) resulting in mRNA cleavage and degradation. This mechanism, distinct from DNA containment (DNAc) and contact unmodified antisense DNA biotechnology (CUADb), forms the basis for RNAi-mediated pest control strategies.

The foundation of RNA interference (RNAi) was established by Andrew Fire and Craig Mello’s seminal discovery that the antisense strand within double-stranded RNA (dsRNA) mediates sequence-specific gene silencing—a breakthrough recognized by the 2006 Nobel Prize in Physiology or Medicine [69]. This pivotal work catalyzed extensive biotechnological applications in agricultural and forestry research for plant protection. RNAi operates through the post-transcriptional and translational suppression of gene expression, utilizing dsRNA molecules typically exceeding 200 base pairs in length that are complementary to target genes [70,71]. The mechanism initiates when the RNase III enzyme (Dicer) cleaves long dsRNA into 20–30 nucleotide small interfering RNAs (siRNAs) [12,72,73,74]. These siRNAs subsequently associate with Argonaute-family proteins to form RNA-induced silencing complexes (RISC) for cytoplasmic mRNA degradation, or RNA-induced transcriptional silencing complexes (RITS) for nuclear gene suppression [74,75]. A parallel pathway involves PIWI-interacting RNAs (piRNAs; 23–36 nucleotides), germline-specific molecules that complex with PIWI-subfamily proteins to repress transposable elements through distinct silencing mechanisms [76,77]. Collectively, these RNAi pathways play crucial regulatory roles during insect ontogenesis (Figure 2), ultimately disrupting the biosynthesis of specific proteins through targeted mRNA degradation or translational blockades.

RNAi efficacy exhibits profound taxonomic variation among economically significant insect pests. Coleopterans demonstrate consistently high systemic efficiency [78,79,80], while Orthoptera (e.g., locusts) and Blattodea (cockroaches) generally exhibit robust responses [81,82,83,84]. Its efficiency is variable in Hymenoptera and Hemiptera due to limited systemic spreading in piercing–sucking insects, attributed primarily to deficient RNA-directed RNA polymerase (RdRP) activity [85,86,87]. Lepidopterans and dipterans typically display low susceptibility to RNAi [88,89]. Consequently, commercial RNAi biopesticide development remains largely confined to coleopteran targets [90,91,92], with target mRNAs spanning genes governing development, detoxification, and reproduction [93] (Table 1).

So far, two primary delivery strategies have been developed for RNAi: genetically modified (GM) plants expressing dsRNA, and the topical application of formulated sprayable products [94]. GM approaches undergo protracted regulatory evaluation under biotechnology frameworks, whereas sprayable formulations—classified as biochemical pesticides—offer potential time and cost advantages, with registration timelines dependent on final product composition [95,96]. Currently, few RNAi products are commercially deployed. The landmark SmartStax^®^ PRO corn (US EPA-registered 2017) combines two Bt insecticidal proteins (Cry3Bb1 and Cry34/35Ab1) with DvSnf7 dsRNA targeting vesicle transport in corn rootworm [97,98]. This triple-mode-of-action pyramid achieves 99% suppression of western (*Diabrotica virgifera virgifera*) and northern (*D. barberi*) corn rootworm larvae, though dsRNA-induced mortality manifests slower than Bt toxicity (≥5 days post ingestion) [97]. The targeted Snf7 protein functions within the ESCRT-III pathway governing transmembrane protein sorting and degradation [99,100], with the documented absence of cross-resistance between DvSnf7 RNAi and Bt toxins [101,102,103]. Experimental GM crops demonstrate efficacy against diverse pests including fall armyworm (*Spodoptera frugiperda*) [104], aphids [105], Colorado potato beetle (*Leptinotarsa decemlineata*) [106], and spider mites (*Tetranychus urticae*) [107]. An advantage of the dsRNA approach is that it is possible to create a long chimeric dsRNA, which can be cleaved into a pool of siRNAs targeting conserved sequences in the pest genomes, and target sites’ resistance against such dsRNA preparations is not expected to develop fast.

The first exogenous sprayable dsRNA biopesticide, Calantha™ (ledprona; IRAC Group 35), was commercialized by GreenLight Biosciences in 2023 against the Colorado potato beetle [108,109,110]. Applied at 9.4 g/ha, it induces near-complete feeding cessation within 2–3 days and achieves 90–95% mortality in 1st–2nd instar larvae within 11–26 days. Its operational constraints include strict larval-stage specificity, its necessity for sequential applications, and recommended rotation with insecticides possessing alternate modes of action. Proof-of-concept successes encompass numerous taxa: the brown planthopper (*Nilaparvata lugens*) [111], African sweet potato weevil (*Cylas puncticollis*) [112], tomato pinworm (*Tuta absoluta*) [113], oriental fruit fly (*Bactrocera dorsalis*) [114], cotton mealybug (*Phenacoccus solenopsis*) [115], diamondback moth (*Plutella xylostella*) [116], fall armyworm (*Spodoptera frugiperda*) [117,118], white-backed planthopper (*Sogatella furcifera*) [105,119], desert locust (*Schistocerca gregaria*) [120], and potato psyllid (*Bactericera cockerelli*) [121,122]—though none have advanced beyond the experimental stages.

The development and deployment of sprayable RNAi-based pesticides raise a number of ethical, legal, and social implications that extend beyond the technical considerations of efficacy and delivery [123], including building public trust and acceptance towards spray-on RNAi biopesticides. From the legal point of view, the spray-on RNAi biopesticide presents regulatory classification and harmonization challenges [124,125]. Proponents advocate for regulatory ambiguities to be clarified, and appropriate and standardized science-based risk assessment to be adopted for the evaluation of sprayable RNAi-based pesticides [125]. Some have argued that plant protection product risk assessments should be adapted to evaluate the unique risks associated with spray-on dsRNA-based pesticides [126], while others insist that the products be regulated as another form of genetic engineering [123]. Several countries such as the US, Canada, Europe, New Zealand and Australia regulate RNAi-based pesticides under existing chemical pesticides regulations. However, many other countries have yet to clarify their regulatory approaches for the technology [124,127].

Despite the promising efficacy of dsRNA technology, there are main challenges restricting this adoption. Target gene selection lacks consensus due to compensatory cellular responses including protein stability, functional redundancy among paralogs, and transcriptional feedback loops [128,129,130]. Proteins with long half-lives may persist even after mRNA levels are significantly reduced, delaying or diminishing the observable phenotypic effects [131]. Gene expression levels and functional redundancy also influence RNAi outcomes: highly expressed genes may require higher doses of dsRNA [132,133]. In contrast, compensatory genes may mitigate the effects of silencing a single target gene [134]. Additionally, compensatory feedback mechanisms may arise in response to gene knockdown. For instance, Willow et al. [135] reported that the RNAi-mediated silencing of the *αCOP* gene in *Brassicogethes aeneus* (Coleoptera: Nitidulidae) led to the post-treatment overexpression of the same gene. This mechanism may undermine the effectiveness of RNAi applied to insects. Therefore, one strategy is to select genes that do not have associated compensatory genes or redundant pathways [136]. Efficacy varies substantially with developmental stage, tissue type, and delivery method. The Spray-Induced Gene Silencing (SIGS) paradigm suffers mechanistic ambiguity—whether dsRNA uptake occurs primarily via integument penetration or oral ingestion and remains unresolved, confounding optimization efforts [137]. Off-target effects may arise from as few as 15 contiguous base matches [138,139] or sequence-independent immune activation [140,141], compounded by formulants (nanocarriers and adjuvants) potentially enhancing non-target exposure [103,142]. Rapid environmental degradation occurs via microbial nucleases in soil/phyllosphere, UV radiation, and precipitation runoff [143,144], with insect-derived nucleases in hemolymph (e.g., *Spodoptera litura* and *Manduca sexta*) and saliva (*Acyrthosiphon pisum*) degrading dsRNA within 0.3–3 h and interfering with robust insecticidal effects [85,145,146]. The environmental safety of the two technologies (CUADb and RNAi) is well presented in the review article Oberemok et al. [16].

While dsRNA production costs have decreased through cell-free platforms like GreenLight’s enzymatic NMP→NTP→dsRNA pipeline [47,48,49,147] and in vivo systems where phi6 polymerase complexes produce target dsRNA molecules inside *Pseudomonas* cells [50], dsRNA is not publicly available, and the affordable price appears dubious. The MEGAscript™ RNAi Kit (Thermo Scientific, Waltham, MA, USA) allows for the production of dsRNA at a cost of ∼$3000 USD for 10 mg. A third option is the production of dsRNA through fermentation. In this process, the dsRNA is synthetized in transgenic cells. The latter process is expected to become the most available and cost-effective method for the large-scale production of dsRNA in the laboratory setting, with target costs near $4 USD/ 1 g [148]. Still, these prices are yet to become commercially feasible, and custom orders for in vitro transcription constructs up to 500 bp in size are still billed at ≥$500 USD for 10 mg [149]. RNA biocontrols remain challenged by their slower lethality and shorter residual activity than conventional insecticides. Sprayable dsRNA offers theoretical advantages over GM crops but requires a deeper mechanistic understanding of RNAi pathways and environmental safety validation. Also, novel delivery systems and chemical modifications of dsRNA may substantially enhance the implementation of RNAi technology in plant protection practice.

## 4. Conclusions

Although both CUAD biotechnology and double-stranded RNA technology utilize nucleic acids as active ingredients, they represent fundamentally distinct insecticidal approaches with divergent mechanisms and applications [9]. Oligonucleotide insecticides employ short, unmodified antisense DNA fragments delivered mainly via contact, operating through the DNA containment (DNAc) mechanism to degrade target ribosomal RNAs in pests. The deoxyribose backbone of DNA fragments confers greater resistance to environmental hydrolysis than their RNA-based counterparts [150], while their compact size (~11 nucleotides) enables exceptional targeting precision. Ribosomal RNA—constituting approximately 80% of cellular RNA—serves as the optimal target due to its abundance and metabolic significance [8,11,17,20], with the current efficacy being most pronounced against sap-feeding sternorrhynchans [8,33]. In contrast, RNA biocontrols utilize longer double-stranded RNA molecules delivered through either contact or oral routes, functioning via the RNA interference (RNAi) pathway to degrade messenger RNAs. The ribose backbone of these molecules increases susceptibility to environmental hydrolysis [151] while their extended length (typically >200 bp) and subsequent enzymatic dicing into smaller fragments complicate selectivity control, necessitating careful risk assessments for non-target organisms [10,93,152,153]. Presently, dsRNA insecticides demand optimized design algorithms and expanded target pest spectra, whereas CUADb-based oligonucleotide insecticides primarily require broader validation across additional pest groups [16]. Of note, CUADb-based insecticides and RNAi insecticides could complement each other’s action and be used in complex formulations for wide range of pests (for example, simultaneously against hemipterans and coleopterans). Also, targeting different natural mechanisms (DNAc and RNAi) will avoid the fast occurrence of resistance to novel nucleic acid-based insecticides.

Both classes benefit from cost-effective synthesis: liquid-phase phosphoramidite methods for DNA oligonucleotides [25,31] and cell-free enzymatic production for dsRNA [147]. Notably, while RNAi mechanisms were characterized years before practical dsRNA insecticides emerged, the DNAc mechanism was discovered following persistent applied research with antisense oligodeoxyribonucleotides in pest control. These innovative technologies will likely demonstrate optimal efficacy against specific pest taxa, offering extended utility through careful design and the potential for synergistic integration in multi-target formulations. Their favorable safety profiles, compared with conventional chemical insecticides for non-target organisms and ecosystems—combining high selectivity, rapid environmental biodegradation, and innovative mechanisms—position nucleic acid-based insecticides as indispensable biomolecules for sustainable agriculture’s future.

## Figures and Tables

**Table 1:** Comparative overview of CUAD biotechnology and dsRNA technology, highlighting differences in effectors, mechanisms, synthesis methods, and pest targets.

Key Features	CUAD Biotechnology (‘Genetic Zipper’ Technology)	dsRNA Technology
Effector	ssDNA	dsRNA
Mechanism	DNA containment First step: rRNA arrest and its hypercompensation; second step: rRNA degradation	RNA interference First step: processing of dsRNA into siRNA; second step: mRNA degradation
Mode of action	ssDNA:rRNA	ssRNA:mRNA
Nuclease involved	DNA(-RNA hybrid)-guided RNase (such as RNase H1)	Argonaute
Synthesis	Liquid-phase/solid-phase oligonucleotide synthesis based on phosphoramidite chemistry [25,31]	Large-scale cell-free biomanufacturing (cell-free dsRNA production) [47,48,49] and utilization of bacteriophage phi6 for the production of high-quality dsRNA molecules [50]
Target pests with best outcome	Hemiptera: Sternorrhyncha	Coleoptera: Chrysomeloidea, Tenebrionoidea

## Data Availability

No new data were created or analyzed in this study. Data sharing is not applicable to this article.
